# Gallic Acid Enhances the Efficacy of BCR::ABL1 Tyrosine Kinase Inhibitors in Chronic Myeloid Leukemia through Inhibition of Mitochondrial Respiration and Modulation of Oncogenic Signaling Pathways

**DOI:** 10.3390/ijms25147958

**Published:** 2024-07-21

**Authors:** Wei Xiang, Colin Sng, Yi-Hui Lam, Ze-Hui Kok, Yeh-Ching Linn, Soek-Ying Neo, Yin-Yin Siew, Deepika Singh, Hwee-Ling Koh, Charles Chuah

**Affiliations:** 1 Department of Haematology, Singapore General Hospital, National Cancer Centre Singapore, Singapore 169608, Singapore; 2 Department of Pharmacy and Pharmaceutical Sciences, Faculty of Science, National University of Singapore, Singapore 117543, Singapore; 3 Program in Cancer and Stem Cell Biology, Duke-NUS Medical School, Singapore 169857, Singapore

**Keywords:** gallic acid, *Leea indica*, mitochondrial respiration, BCR::ABL1, CML

## Abstract

While BCR::ABL1 tyrosine kinase inhibitors have transformed the treatment paradigm for chronic myeloid leukemia (CML), disease progression and treatment resistance due to BCR::ABL1-dependent and BCR::ABL1-independent mechanisms remain a therapeutic challenge. Natural compounds derived from plants have significantly contributed to cancer pharmacotherapy. This study investigated the efficacy of an active component of *Leea indica*, a local medicinal plant, in CML. Using high-performance liquid chromatography–electrospray ionization–mass spectrometry, a chemical constituent from *L. indica* extract was isolated and identified as gallic acid. Commercially obtained gallic acid was used as a chemical standard. Gallic acid from *L. indica* inhibited proliferation and induced apoptosis in CML cell lines, as did the chemical standard. Furthermore, gallic acid induced apoptosis and decreased the colony formation of primary CML CD34^+^ cells. The combination of isolated gallic acid or its chemical standard with BCR::ABL1 tyrosine kinase inhibitors resulted in a significantly greater inhibition of colony formation and cell growth compared to a single drug alone. Mechanistically, CML cells treated with gallic acid exhibited the disruption of multiple oncogenic pathways including ERK/MAPK, FLT3 and JAK/STAT, as well as impaired mitochondrial respiration. Rescue studies showed that gallic acid is significantly less effective in inducing apoptosis in mitochondrial respiration-deficient ρ^0^ cells compared to wildtype cells, suggesting that the action of gallic acid is largely through the inhibition of mitochondrial respiration. Our findings highlight the therapeutic potential of *L. indica* in CML and suggest that gallic acid may be a promising lead chemical constituent for further development for CML treatment.

## 1. Introduction

Chronic myeloid leukemia (CML) is a hematological malignancy characterized by the presence of the fusion gene BCR::ABL1, which encodes a constitutively active tyrosine kinase upstream of essential cell survival and proliferation pathways [1]. BCR::ABL1 tyrosine kinase inhibitors (TKIs) such as imatinib, have revolutionized the treatment of CML such that patients diagnosed and treated during chronic phase CML (CP-CML) are expected to have an excellent clinical response and normal life expectancy [2]. However, the prognosis for CML after progression to the more aggressive blast phase is dismal, with a median survival of between 7 and 11 months [3]. Despite the introduction of more potent BCR::ABL TKIs, the majority of patients with CML in the blast phase are resistant to treatment or relapse after an initial response. Allogeneic stem cell transplant is the only therapy that provides long-term remission, but transplant-related mortality and morbidity are high. The lack of a suitable donor also precludes many patients from being eligible for a transplant [3]. There is therefore a need to explore alternative therapeutic strategies for CML.

Herbal medicine has been used extensively in developing countries for the treatment of various diseases, and traditional herbs contain bioactive chemicals that can be modified and further developed into conventional therapeutics [4]. Naturally occurring compounds constitute a significant proportion of conventional chemotherapeutics including taxanes (e.g., paclitaxel), vinca alkaloids (e.g., vincristine) and topoisomerase inhibitors (e.g., etoposide) [5,6,7]. *Leea. indica* (Burm. f.) Merrill is a Southeast Asian medicinal plant used as treatment for a number of medical conditions [8,9] and is reported to have anti-bacterial, antioxidant and anti-inflammatory properties [10]. Studies have shown that *L. indica* also displays anti-cancer activity [11,12,13]. We had also observed a positive clinical response to a decoction of *L. indica* leaves in an advanced-phase CML patient who was resistant to dasatinib, a second-generation BCR::ABL1 TKI. The fractions from *L. indica* extract have been reported to inhibit proliferation [12,13,14] and induce apoptosis in a number of cancer cell lines [12,13]. We subsequently isolated a chemical constituent from *L. indica* leaves, which was identified as gallic acid by nuclear magnetic resonance (NMR) spectroscopy and liquid chromatography mass spectrometry (LC-MS) analyses [8]. Gallic acid has been reported to exhibit anti-cancer activity in solid and liquid cancers, such as prostate cancer [15], breast cancer [16], non-small cell lung cancer [17] and leukemia [18], through a range of mechanisms such as histone deacetylase inhibition [15], p53/Bax overexpression [16], the modulation of oncogenic pathways [17,19,20] and the inhibition of mitochondrial respiration [18].

In this study, we investigated the effects of isolated gallic acid, denoted herein as GI, on CML cells and primary stem/progenitor cells. To determine if isolated gallic acid is equipotent to the commercial standard, we investigated the effects of commercial standard gallic acid, denoted herein as GA, on CML. Our results show that the efficacies of GI and GA in CML are similar. In addition, gallic acid acts through inhibiting mitochondrial respiration as well as a number of oncogenic signaling pathways. Our study suggests that these mechanisms are distinct processes and are required for gallic acid to be efficacious in CML.

## 2. Results

### 2.1. Gallic Acid Decreases Proliferation and Induces Apoptosis in CML Cells

**Figure 1:**
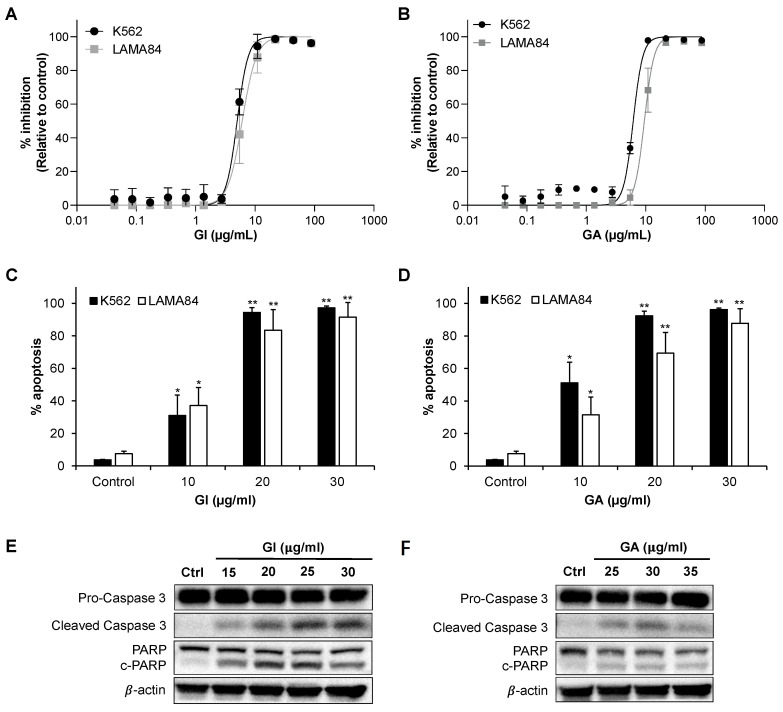
Gallic acid isolated from *L. indica* (GI) and gallic acid commercial standard (GA) inhibit cell proliferation and induce apoptosis in CML cell lines in a similar manner. (**A**,**B**) Drug response curve of K562 and LAMA84 cells to GI and GA. (**C**,**D**) GI and GA significantly induce apoptosis in CML cells. Treatment was 72 h. (**E**,**F**) Western immunoblotting of pro-apoptotic proteins shows an increase in both caspase 3 and PARP cleavage after 72 h of GI and GA treatment, respectively, in LAMA84 cells. β-actin serves as a loading control. Data are representative of at least three independent triplicates. * indicates *p* < 0.05 and ** indicates *p* < 0.01.

K562 and LAMA84 cells were treated with increasing concentrations of GI and GA for 72 h, and proliferation was determined. GI and GA inhibited proliferation, with IC_50_ values of 5.6 ± 1.9, 7.1 ± 1.6 μg/mL (K562) and 6.7 ± 1.5, 9.0 ± 1.8 μg/mL (LAMA84), respectively (average ± standard deviation; Figure 1A,B). Apoptotic morphological changes after treatment with GI and GA, as evidenced by cell shrinkage, membrane blebbing and a loss of membrane integrity, were observed, (Supplementary Figure S1). We showed that GI and GA induce dose-dependent apoptosis (as indicated by Annexin V-FITC+/7-AAD- and Annexin V-FITC+/7-AAD+ populations) in K562 and LAMA84 cells (Figure 1C,D and Supplementary Figure S2). An immunoblotting analysis of essential molecules involved in apoptotic pathways showed that GI and GA led to an increase in cleaved caspase 3 and cleaved PARP (Figure 1E,F and Supplementary Figure S3).

### 2.2. Gallic Acid Decreases Colony Formation and Survival in Primary CML CD34^+^ Cells

**Figure 2:**
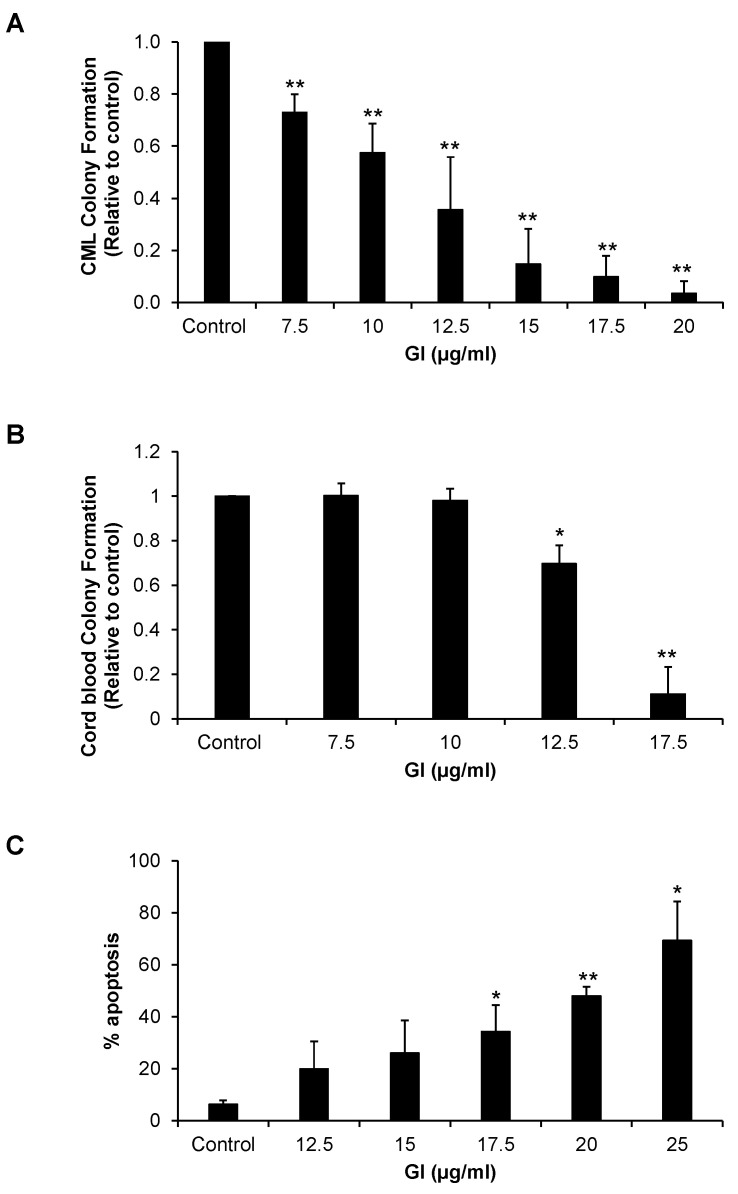
GI decreases colony formation and induces apoptosis in CML stem/progenitor cells. (**A**,**B**) GI inhibits the colony formation of primary CML CD34^+^ cells in a dose-dependent manner and to a greater extent than normal cord blood CD34^+^ cells. Colonies were counted under a microscope and the results are presented as relative to the control. Four CML patient samples and three healthy cord blood samples were included in this study. (**C**) GI also induces apoptosis dose-dependently in primary CML cells after 72 h. All data were conducted in independent triplicates. * indicates *p* < 0.05 and ** indicates *p* < 0.01.

We next explored the effects of GI on primary CML stem/progenitor cells. Characteristics of CML stem/progenitor cells include proliferation and multi-lineage differentiation, which can be determined through a colony formation assay. GI at concentrations of 7.5 μg/mL to 20 μg/mL decreased colony formation in a dose-dependent manner (Figure 2A and Supplementary Figure S4). In cord blood CD34^+^ cells, a decrease in colony formation was only seen with GI at concentrations of 12.5 μg/mL and 20 μg/mL, suggesting that gallic acid is selective in inhibiting CML colony formation (Figure 2B and Supplementary Figure S4). GI also induced apoptosis in primary CML CD34^+^ cells in a dose-dependent manner (Figure 2C).

### 2.3. Gallic Acid Is Synergistic with BCR::ABL1 TKIs in CML Cell Lines and Primary CML Cells

**Figure 3:**
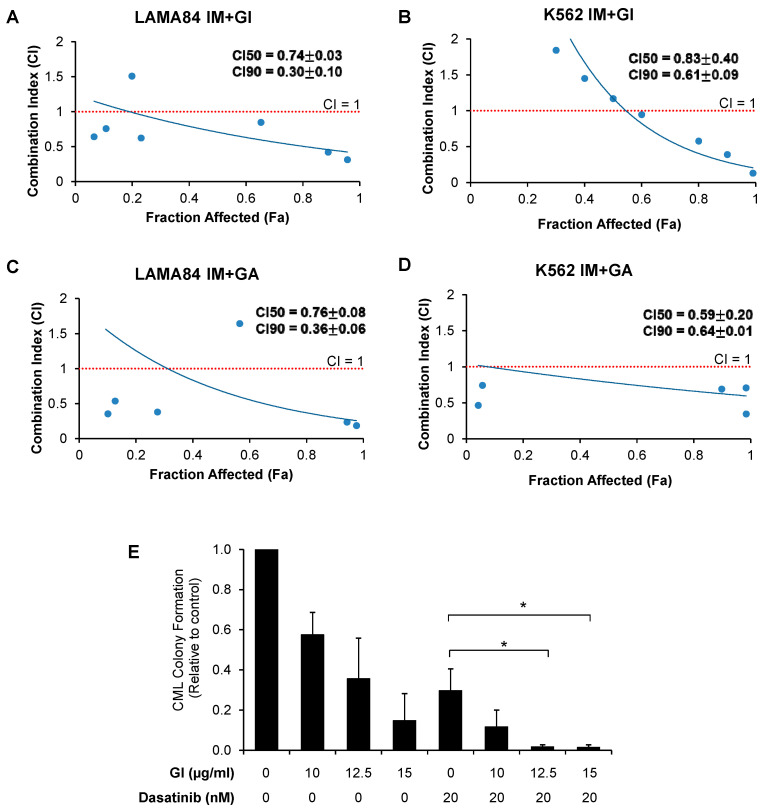
GI and GA exhibit synergistic inhibitory effects with BCR::ABL1 TKIs in both CML cell lines and stem/progenitor cells. (**A**–**D**) Combination index (CI) was calculated using the method of Chou and Talalay and CI < 1 indicates synergism, CI = 1 is additive and CI > 1 suggests antagonism. GI and GA demonstrated synergy with imatinib in both LAMA84 and K562 cell lines with CI < 1 at both 50% and 90% growth inhibition. Data are representative of the mean and SD of at least three independent experiments. (**E**) GI significantly enhanced with the inhibitory effect of dasatinib on the colony formation of CML CD34^+^ (n = 3). In total, 20nM of dasatinib was used. All data were conducted in independent triplicates. * indicates *p* < 0.05.

To examine if GI and GA exhibit synergy with BCR::ABL1 TKIs, we performed combination studies using the first-generation TKI, imatinib, the second-generation TKI, dasatinib, and the third-generation TKI, ponatinib. Compared to imatinib, dasatinib exhibits greater in vitro potency and is active against most imatinib-resistant BCR::ABL1 kinase mutations except the T315I mutation. Ponatinib is active against BCR::ABL1 kinase mutations including the T315I mutation. Imatinib and ponatinib were used in experiments for CML cell lines. Dasatinib was used in experiments for patient samples, which were imatinib-resistant (Supplementary Table S1). We treated the CML cell lines with GI and GA in combination with the BCR::ABL1 TKIs based on methods proposed by Chou and Talalay [21] and measured proliferation. GI was synergistic with imatinib in K562 and LAMA84 cells with a combination index (CI) < 1 at 50% inhibition (Figure 3A,B and Supplementary Table S2). Similarly, GA demonstrated synergy with imatinib in K562 and LAMA84 cells with a CI < 1 at 50% inhibition (Figure 3C,D and Supplementary Table S2). Similar to imatinib, we also showed that the combination of GI or GA with ponatinib exhibited synergistic effects in suppressing proliferation in K562 and LAMA84 cells (Supplementary Figure S5).

We next determined if gallic acid enhanced the efficacy of BCR::ABL1 TKI in primary CML stem/progenitor cells. As the cells were isolated from CML patients who were imatinib-resistant, we used the second-generation BCR::ABL1 TKI, dasatinib. Consistent with our observation with the CML cell lines, the combination of dasatinib and GI significantly decreased primary CML colony formation as compared to dasatinib alone (Figure 3E and Supplementary Figure S6).

### 2.4. Gallic Acid Modulates Oncogenic Pathways in CML

**Figure 4:**
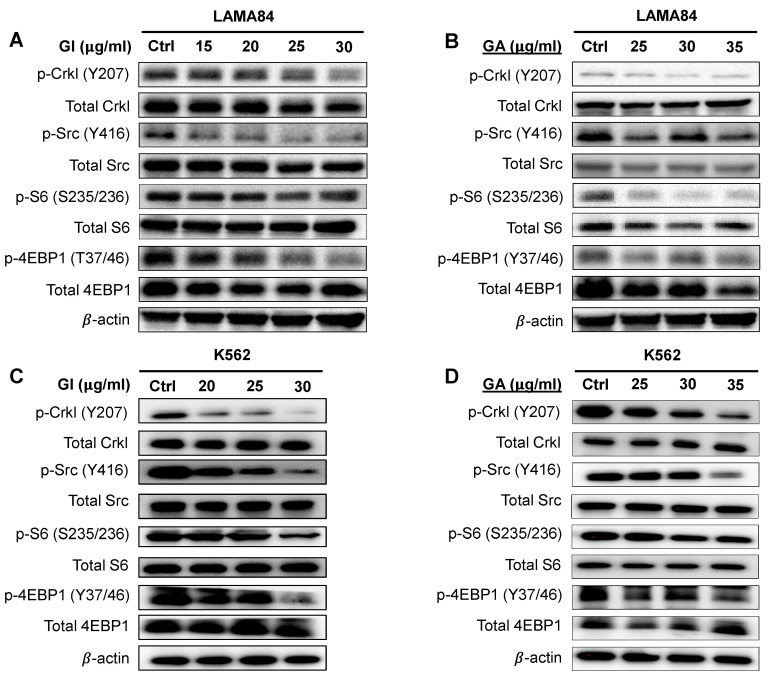
The inhibitory effects of GI and GA in BCR::ABL1-mediated pathways in CML cells. GI (**A**,**C**) and GA (**B**,**D**) decrease the phosphorylation of SRC, CrkL, S6 ribosomal protein and 4EBP-1 in LAMA84 and K562 CML cells after 24 h of treatment. β-actin serves as a loading control. Data are representative of at least three independent triplicates.

To investigate the mechanism of action of gallic acid in CML, we first explored the effect of GI and GA on the BCR::ABL1, SRC and mTOR pathways in LAMA84 cells. Phosphorylation of the adaptor protein CRKL, which is used to assess BCR::ABL1 kinase activity, was reduced in a dose-dependent manner with treatment with GI and GA. The phosphorylation levels of SRC (Y416) were decreased, as were those of the proteins involved in the mTOR pathway, such as S6 and 4-EBP1 (Figure 4A,B and Supplementary Figure S7). We further showed that the inhibitory effects of GI and GA upon oncogenic signaling pathways were observed in K562 cells (Figure 4C,D and Supplementary Figure S7). These results show that gallic acid exhibits anti-cancer effects via the modulation of oncogenic signaling pathways.

**Figure 5:**
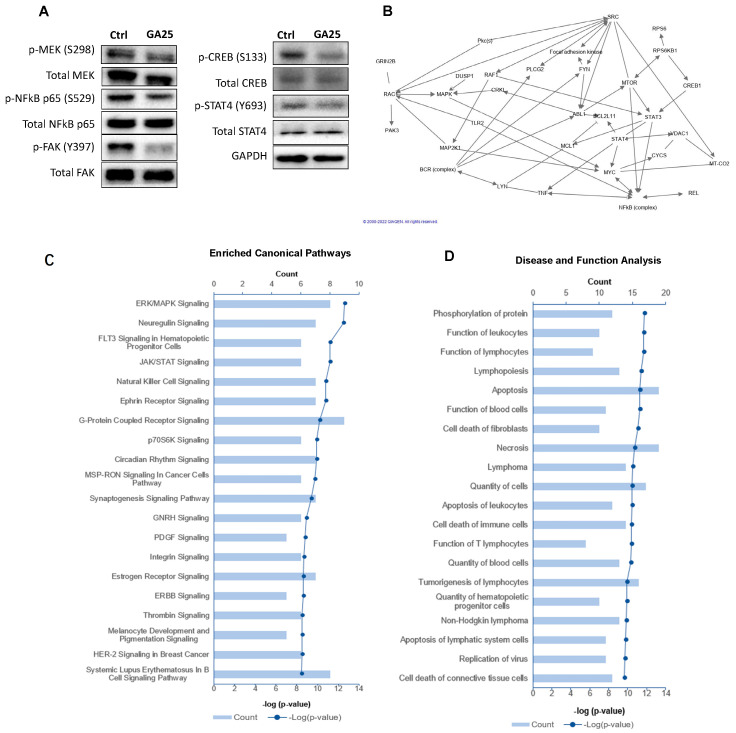
GA is predicted to be involved in multiple signaling pathways in LAMA84 cells. Phosphoprotein profiling was conducted using LAMA84 lysates collected after GA treatment at 25 μg/mL for 24 h. Phosphoprotein profiling results were analyzed using Ingenuity Pathway Analysis (IPA) software 22.0.1. (**A**) Western immunoblotting of MEK and FAK were conducted as secondary validation for phosphoprotein profiling results in LAMA84 cells. GAPDH serves as a loading control. (**B**) IPA predicted networks involving top hits from phosphoprotein profiling and molecules involved in BCR::ABL1, SRC and mTOR pathways to elucidate further connections between molecules. (**C**) Canonical pathway analysis predicted by IPA to be affected by GA treatment based on the number of molecules from the phosphoprotein profiling results involved in the respective pathways, and the significance is shown in –log(*p*-value). (**D**) Diseases and functions affected by GA treatment were predicted using IPA software with the same set of molecules, and significance is shown in –log(*p*-value).

We next conducted phosphoprotein profiling using the Phospho Explorer Array PEX100 kit that covers 1318 proteins in 30 different oncogenic signaling pathways to explore changes in phosphorylation with GA treatment in LAMA84 cells. Proteins with a reduction of more than twofold in phosphorylation with treatment were identified (Supplementary Table S3). The majority of identified proteins affected by GA are those involved in transcription signaling (e.g., REL, CREB, STAT2, CREB and NFkB) and the cell cycle. Representative phosphoproteins were then selected and validated by immunoblotting in LAMA84 and K562 cells, such as p-MEK (S298), p-NFkB p65 (S529), p-FAK (Y397), p-DUSP1/MKP1 (S359), p-CREB (S133) and p-STAT4 (Y693) (Figure 5A and Supplementary Figure S8), demonstrating a concordance rate of 75%. The list of validated altered proteins analyzed by Ingenuity Pathway Analysis (IPA) revealed more than 10 significant networks (Figure 5B). A total of 14 enriched canonical pathways were identified by applying the -log (*p*-value) > 8 (Figure 5C). The top pathways identified include ERK/MAPK (*p*-value = 2.51 × 10^−13^), FLT3 (*p*-value = 6.31 × 10^−12^) and JAK/STAT (*p*-value = 6.31 × 10^−12^) signaling pathways, all of which play significant roles in CML cell survival and apoptosis. The analysis of common diseases and functions demonstrates that this set of molecules is strongly associated with leukocyte function (*p*-value = 2 × 10^−12^), lymphocyte function (*p*-value = 2.2 × 10^−12^) and lymphopoiesis (*p*-value = 3.8 × 10^−12^) (Figure 5D).

### 2.5. Gallic Acid Inhibits Mitochondrial Respiration in CML

**Figure 6:**
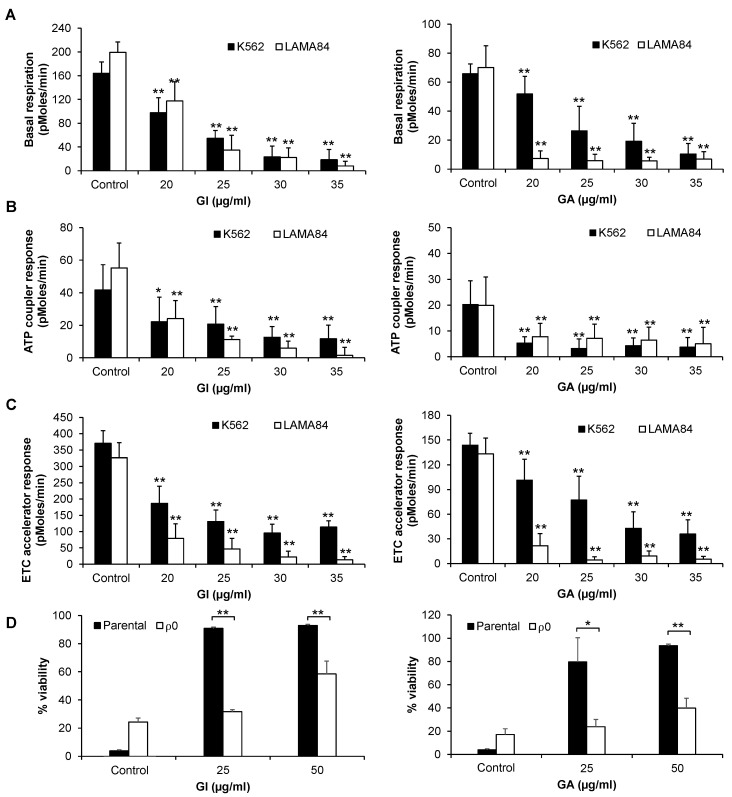
GI and GA are ineffective in mitochondrial respiration-deficient CML ρ0 cells. (**A**–**C**) GI and GA significantly decrease the basal respiration, ATP coupler response and ETC accelerator response in K562 and LAMA84 cells. (**D**) The pro-apoptotic effects of GI and GA are significantly abolished in mitochondrial respiration-deficient LAMA84 ρ^0^ cells. All experiments were conducted in independent triplicates. * indicates *p* < 0.05 and ** indicates *p* < 0.01.

Gallic acid has been shown to inhibit mitochondrial respiration in acute myeloid leukemia [18]. Given the importance of mitochondrial respiration in myeloid leukemia [22], we explored the effect of GI and GA on mitochondrial respiration in CML cell lines. Both GI- and GA-treated K562 and LAMA84 cells exhibit a significant dose-dependent reduction in basal respiration (Figure 6A). Our results further show that with the addition of oligomycin, GI and GA induce a significant decrease in ATP-coupled mitochondrial respiration (indicating minimal respiration) as compared to the control in both cell lines (Figure 6B). The ETC accelerator response, which indicates maximal respiration, was also decreased upon treatment with GI and GA in response to the addition of carbonyl cyanide-4 (trifluoromethoxy) phenylhydrazone (FCCP) (Figure 6C). To confirm that mitochondrial respiration is the target of gallic acid, we investigated the effects of GI and GA in mitochondrial respiration-deficient ρ^0^ cells established in our laboratory [23]. We showed that the level of apoptosis is significantly decreased in the ρ^0^ LAMA84 cells as compared to the parental LAMA84 cells upon treatment with GI and GA (Figure 6D). Taken together, our results indicate that the action of gallic acid in CML is largely through inhibiting mitochondrial respiration.

## 3. Discussion

Plants contain active chemical constituents and are important sources of phytochemicals that have great promise for treating a variety of diseases, including cancer [24]. Plant-derived natural compounds have been used extensively in pharmacotherapy in cancer, with approximately 60% of clinical chemotherapeutic agents originating from plants [25,26]. Although *L. indica* has been used as a form of traditional medicine in treating cancer [27], the chemical constituents responsible for its anti-cancer properties are not well understood.

By isolating and analyzing the *L. indica* ethyl acetate fraction using HPLC-ESI-MS, we previously reported that *L. indica* is a source of a wide range of phenolic contents, which might explain the diverse pharmacological activities of *L. indica* [8,9]. Compared to other *L. indica* chemical constituents, gallic acid displayed anti-cancer activity [14], and thus, its effects and mechanism of action were further investigated in the context of CML. Our results extend the observations of a previous study [20] by showing that gallic acid induces apoptosis in 2 CML cell lines, K562 and LAMA84. Through the utilization of NMR and LC-MS analyses, we confirmed the structural identity of isolated gallic acid by comparing it against a commercial standard [8]. Biologically, we demonstrated no discernible difference between both compounds, which is consistent with the chemical analyses confirming their identical nature. We have shown that both isolated gallic acid and its commercial standard inhibit proliferation and induce apoptosis with similar potencies and significantly augment the efficacy of BCR::ABL1 TKIs in CML cell lines.

Disease relapse upon BCR::ABL1 TKI treatment discontinuation or the development of treatment resistance in CML can occur due to the persistent leukemia stem/progenitor cell population [28]. We show that isolated gallic acid is effective in inducing apoptosis and inhibiting the colony formation of primary CML CD34^+^, suggesting that isolated gallic acid targets the CML stem/progenitor population. Previous studies on the anti-cancer effects of gallic acid have focused primarily on differentiated cancer cells [15,16,17,19,29]. A recent study suggested that gallic acid not only inhibits differentiated cancer cells but also targets cancer stem cells [30]. Our findings corroborate this observation and highlight the anti-leukemia stem cell property of gallic acid. Leukemia stem cells are responsible for disease initiation, maintenance and relapse [31]. A drug that can target stem/progenitor cells in CML, in addition to differentiated cancer cells, would represent a major advancement in the treatment strategy, offering the potential for more effective and durable clinical responses.

The significantly enhanced combinatory effect of isolated gallic acid and dasatinib further confirms that gallic acid is effective in inhibiting the proliferation of TKI-resistant primary CML stem/progenitor cells. Residual stem/progenitor cells are largely responsible for leukemia relapse. To the best of our knowledge, this is the first report that demonstrates that gallic acid inhibits the proliferation of drug-resistant stem/progenitor cells in leukemia. We also noted that isolated gallic acid at 17.5 µg/mL inhibits the colony formation of normal cord blood CD34^+^ in a similar manner as CML, suggesting a narrow therapeutic window. Further efforts to improve the efficacy and reduce the toxicity of gallic acid will be required.

The understanding of the molecular target and mechanism of action is essential for drug discovery and development. Our study has shown that gallic acid inhibits oncogenic pathways such as BCR::ABL1, SRC and mTOR. As pathways induced by SRC kinases and PI3K/mTOR are also regulated by BCR::ABL1 activity [32,33], we hypothesize that the inhibition of SRC and mTOR might be the consequence of BCR::ABL1 inhibition. In agreement with our hypothesis, IPA analysis based on our phosphoprotein profiling data shows that ERK/MAPK and JAK/STAT, which are also downstream of BCR::ABL1, are the top signaling pathways negatively affected in gallic acid-treated CML cells. Gallic acid has previously been reported to inhibit BCR::ABL1 tyrosine phosphorylation in a similar manner as imatinib [20]. In addition, gallic acid inhibits EGFR tyrosine phosphorylation in non-small cell lung and colon cancer cells [19,29]. While the mechanism of action of gallic acid in cancer cells is not universal but cancer cell type-specific [15,16,17,18], our findings suggest that the anti-leukemic activity of gallic acid is in part due to its ability in targeting multiple tyrosine kinase pathways.

Our data indicate that mitochondrial respiration is a target of gallic acid in CML cells. Both isolated gallic acid and its commercial standard disrupt mitochondrial respiration via decreasing the basal respiration, ATP coupler response and ETC accelerator response. Furthermore, we showed that mitochondrial respiration-deficient LAMA84 ρ^0^ cells are less sensitive to isolated gallic acid and its commercial standard, suggesting that gallic acid acts on CML via the inhibition of mitochondrial respiration. Mitochondrial respiration is a promising therapeutic target for leukemia, as leukemia cells, particularly leukemic stem cells, are more dependent on mitochondrial respiration than their normal hematopoietic counterparts for survival [34,35]. We previously showed that the inhibition of mitochondrial respiration is selective and effective in targeting BP-CML [23].

In conclusion, our findings show that isolated gallic acid and its commercial standard are active against both stem/progenitor and differentiated CML cells and synergize with BCR::ABL1 TKIs through the inhibition of mitochondrial respiration and oncogenic signaling pathways. This previously unreported dual mechanism of action for gallic acid in CML provides an attractive therapeutic strategy, particularly for BCR::ABL1 TKI-resistant disease. Based on our findings, we will determine the efficacy and pharmacokinetics of gallic acid using CML patient-derived xenograft mouse models. Successful completion will set the stage for early-phase clinical trials.

## 4. Materials and Methods

### 4.1. Primary Cells

Primary peripheral blood or bone marrow samples were obtained from CML patients from Singapore General Hospital after obtaining written informed consent in accordance with institutional review board-approved protocols. Patient clinical information is in Supplemental Table S1. Cord blood samples were obtained from the Singapore Cord Blood Bank. Mononuclear cells were isolated with density gradient centrifugation using Histopaque-1077 from, followed by CD34^+^ cells purification using the CD34 MicroBead kit (Miltenyi Biotec, Bergisch Gladbach, Germany). We cultured CD34^+^ primary cells using a complete medium (Life Technologies, Carlsbad, CA, USA), supplemented with 200 pg/mL of the stem cell factor, 200 pg/mL of the granulocyte-macrophage colony-stimulating factor, 200 pg/mL of macrophage inflammatory protein-1α, 1 ng/mL of the granulocyte colony-stimulating factor, 50 pg/mL of the leukemia inhibitory factor and 1 ng/mL of interleukin 6. The cells were cultured in the aforementioned medium for 24 h before being subjected to the respective assays.

### 4.2. Cell Lines

K562 and LAMA84 human CML cell lines (kind gift from Dr Junia Melo) were maintained in suspension in RPMI 1640 medium (Gibco, Thermo Fisher Scientific, Waltham, MA, USA), supplemented with 10% fetal bovine serum, 2 mM L-glutamine (Hyclone, Logan, UT, USA), 100 U/mL penicillin and 100 μg/mL streptomycin (Gibco, Thermo Fisher Scientific, USA), at 37 °C in 5% CO_2_. LAMA84 ρ^0^ cells, which were established in our laboratory [23] and used to identify if mitochondrial respiration is essential for the action of the drugs and compounds that were tested. LAMA84 ρ^0^ cells were maintained in suspension in RPMI 1640 medium containing 10% FBS, 4 mM L-glutamine, 50 µg/mL uridine and 100 µg/mL sodium pyruvate.

### 4.3. Drugs and Compounds

Imatinib methanesulfonate salt (LC Laboratories, Woburn, MA, USA), ponatinib (LC Laboratories, USA) and dasatinib (LC Laboratories) were dissolved in sterile distilled water and dimethyl sulfoxide (DMSO), respectively. Gallic acid was isolated from *L. indica* leaves using the method previously reported [8]. Briefly, freshly harvested leaves of *L. indica* were cleaned and air-dried. They were blended using a Morries blender (Singapore) and extracted by maceration using 70% methanol for 3 days. The solvent was decanted and the process was repeated twice with fresh solvent each time, for a total of 9 days. The combined 70% methanolic extract was dried, reconstituted in water and further fractionated by liquid–liquid extraction using hexane, dichloromethane and ethyl acetate. The ethyl acetate fraction was subjected to column chromatography, and gallic acid was isolated. The estimated concentration of gallic acid in the fresh leaves is 0.005–0.011% *w*/*w*. Commercial standard gallic acid was purchased from Sigma-Aldrich (St. Louis, MO, USA) and was dissolved in DMSO.

### 4.4. Measurement of Apoptosis

The cells were plated at a 1 × 10^5^/mL density on 12-well-plates and treated with drugs and compounds for 72 h. Cell apoptosis was performed by Annexin V-FITC and 7-aminoactinomycin D (7-AAD) (IM3614; Beckman Coulter, Villepinte, France) staining, as per the manufacturer’s protocol, and was analyzed on BD LSRFortessa. Early apoptosis was defined as Annexin V-FITC+/7-AAD-, while late apoptosis was defined as Annexin V-FITC+/7-AAD+. Total apoptosis was defined as the sum of both Annexin V-FITC+/7-AAD- and Annexin V-FITC+/7-AAD+ populations.

### 4.5. Cell Proliferation Assay and Drug Combination Studies

Cells were plated at a density of 1 × 10^5^ on 96-well-plates and treated with drugs and compounds for 72 h. Cellular proliferation was analyzed by the CellTiter 96 Aqueous One Solution Cell Proliferation assay kit (Promega, Fitchburg, WI, USA), following the manufacturer’s instructions. To determine the combinatory effects of imatinib and isolated gallic acid or its commercial standard, combination studies were designed based on the methods described by Chou and Talalay [21]. The cells were treated with isolated gallic acid or its commercial standard and BCR::ABL1 TKI or in an equipotent constant-ratio combination. The concentrations of each compound and drug were indicated in Supplementary Table S2. The combination indices (CI) at 50% growth inhibition, calculated by the CompuSyn software 1.0 (Biosoft, Grimsby, UK), were used as a guide to quantitatively demonstrate combination effects (synergism, CI < 1; additive effect, CI = 1; antagonism, CI > 1).

### 4.6. Colony-Forming Assay

Primary CD34^+^ cells at 1–5 × 10^3^ were plated in StemMACS^TM^ HSC-CFU complete methylcellulose medium w/o Epo (Miltenyi Biotec, Bergisch, Germany). After two weeks of treatment, the number of colonies was scored. Images were captured with a microscope (Olympus BX53, Tokyo, Japan).

### 4.7. Western Blot (WB) Analysis

After 24 h of treatment, the cells were lysed in 20 mM pH 8.2 Tris-HCl, 150 mM NaCl and 1% Triton X-100, supplemented with cOmplete mini EDTA-free Protease Inhibitor Cocktail (Roche Diagnostics, Mannheim, Germany). Equal amounts of the protein were resolved onto SDS-PAGE gels and analyzed by WB using the primary antibodies (Cell Signalling Technology, Beverly, MA, USA) p-SRC (Y416; #6943S), total SRC (#2108S), p-Crkl (Y207; #3181S), total Crkl (#3182S), p-S6 (S235/236; #2211S), total S6 (#2217S), p-4EBP1 (T37/46; #2855S), total 4EBP1 (#9644S), p-MEK (S298; sc271914), total MEK (sc6250), p-NFkB p65 (S536; #3033S), total NFkB p65 (#8242S), p-FAK (Y397; sc81493), total FAK (sc271126), p-DUSP1/MKP1 (S359; #2857S), total DUSP/MKP1 (#48625S), p-CREB (S133, #9198S), total CREB (#9198S), p-STAT4 (Y693; sc136194) and total STAT4 (sc398228). Proteins were detected using the Immobilon Western (Millipore, Burlington, MA, USA). Images were captured via the Chemidoc XRS+ Imaging System (Bio-Rad Laboratories, Hercules, CA, USA), and quantification was performed using the ImageJ 1.54 (National Institutes of Health, Bethesda, MD, USA) software.

### 4.8. Phospho-Antibody Array

Phosphoprotein profiling was performed using the Phospho Explorer Array PEX100 kit (Full Moon BioSystems Inc., Sunnyvale, CA, USA) according to the manufacturer’s instructions on LAMA84 cells treated with drugs or compounds for 24 h. The samples were labelled with Alexa Fluor 555 streptavidin (S32355; Invitrogen, Carlsbad, CA, USA) and scanned using a GenePix 4000 microarray scanner (Agilent Technologies, Santa Clara, CA, USA).

### 4.9. Oxygen Consumption Rate (OCR) Measurement

After treatment for 24 h, 5 × 10^4^ drug-treated cells/well were resuspended in the XF assay medium (Agilent Technologies) and were seeded in 96-well XF96 well plates coated with BD Cell-Tak (BD Biosciences, Fremont, CA, USA). One hour before performing the OCR assay, the cells were incubated at 37 °C in a CO_2_-free environment. OCR was measured with the Seahorse XF Cell Mito Stress Test Kit (Agilent Technologies, USA) on a Seahorse XFe96 Analyzer (Agilent Technologies, USA) according to the manufacturer’s instructions. Analyses were performed both at basal conditions and after the injection of oligomycin, carbonyl cyanide-p-trifluoromethoxyphenylhydrazone and the antimycin A and rotenone combination.

### 4.10. Data Analysis

All experiments were conducted in triplicate, and the data are presented in the average ± standard deviation, unless stated otherwise. Student’s *t*-test was used to calculate the probability against the control data. Statistical analyses, including one-way and two-way ANOVA for single-drug and combination-drug treatments, were performed using Prism 8.0.1.

## Data Availability

Data are contained within the article.

## Supplementary Materials

The following supporting information can be downloaded at: https://www.mdpi.com/article/10.3390/ijms25147958/s1.
