# Supplementary material for: Gallic Acid Enhances the Efficacy of BCR::ABL1 Tyrosine Kinase Inhibitors in Chronic Myeloid Leukemia through Inhibition of Mitochondrial Respiration and Modulation of Oncogenic Signaling Pathways
Source: Int J Mol Sci. 2024 Jul 21;25(14):7958. doi: 10.3390/ijms25147958 (PMC11276718; doi:10.3390/ijms25147958)
Supplement: Supplementary file 1 [file ijms-25-07958-s001.zip › ijms-3056873-supplementary.pdf]

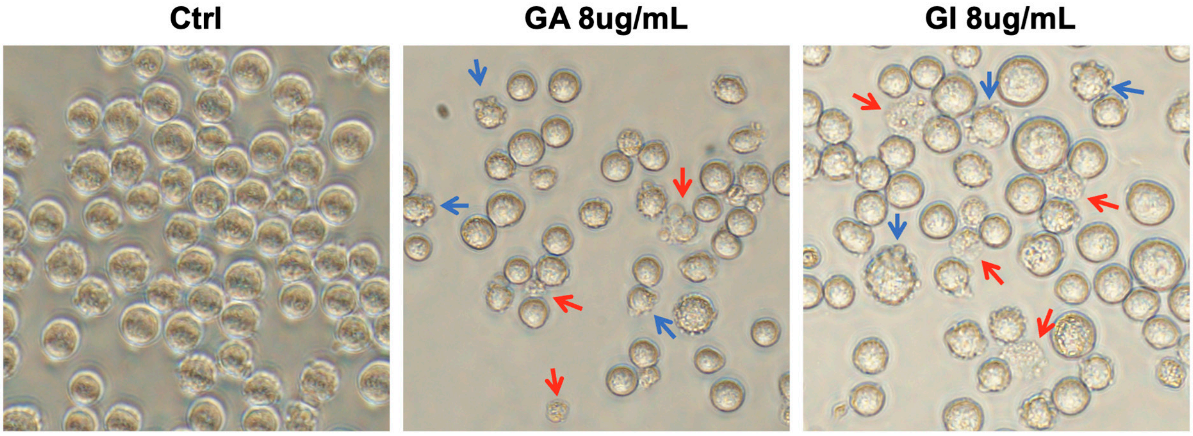

Supplementary Figure S1: K562 morphology changes after GI and GA treatment. Cells were treated with appropriate amounts of drug for 72 h. Blue arrows indicate membrane blebbing and red arrows indicate loss of membrane integrity.

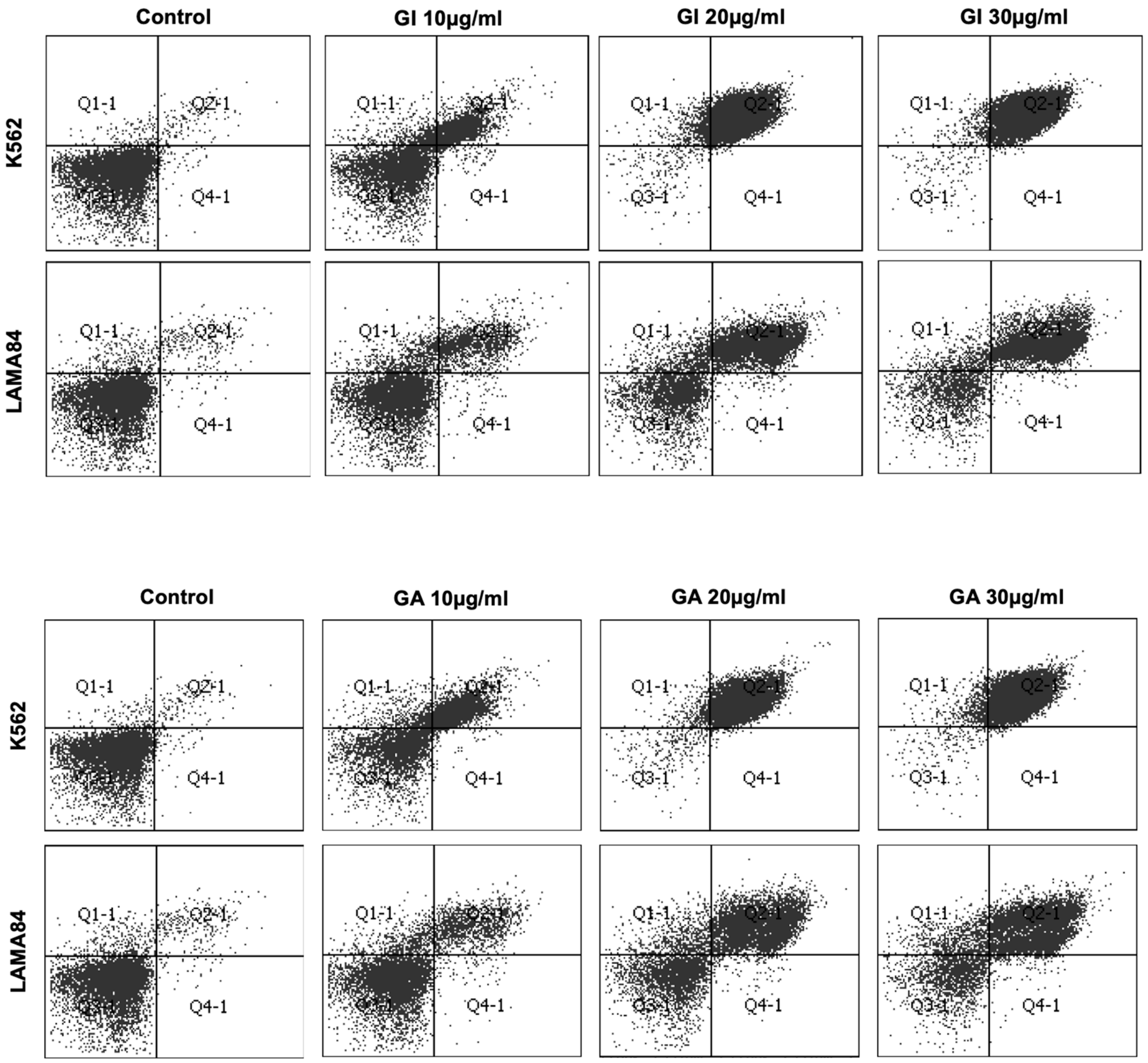

Supplementary Figure S2: GI and GA increase proportion of cells in FITC+/7-AAD- and FITC+/7-AAD+ quadrants in both K562 and LAMA84 cells. Cells were treated with appropriate amounts of drug for 72 h before staining with Annexin V-FITC and 7-AAD. They were then analyzed on the BD Fortessa (x-axis: 7-AAD; y-axis: FITC) with the BDFACSDiva software. Images are representative of three independent replicates.

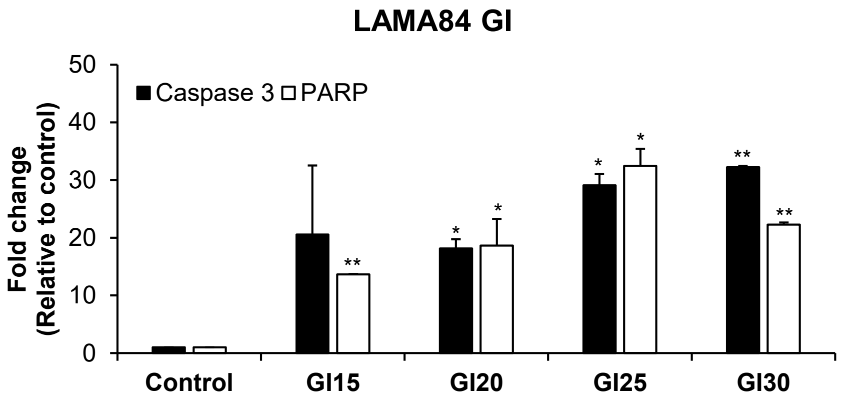

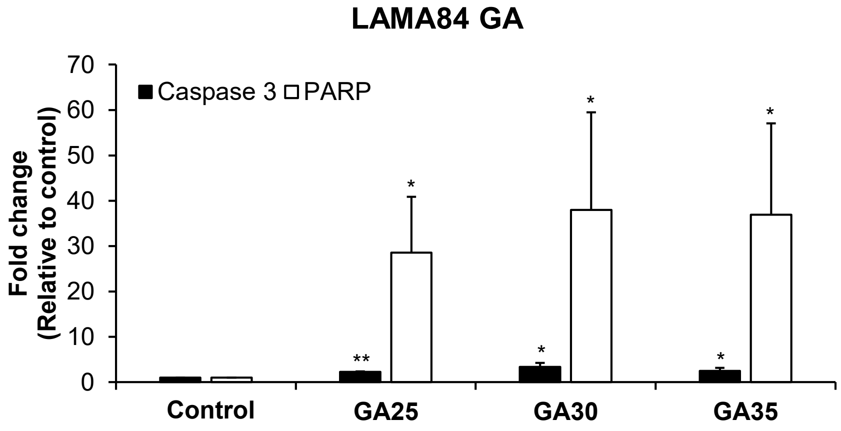

Supplementary Figure S3: Densitometries of cleaved/pro-caspase-3 and cleaved/uncleaved-PARP were obtained via ImageJ and normalized against β-actin. Bar graphs are then plotted using fold change of treated samples against control. (* indicates $p < 0.05$, ** indicates $p < 0.01$)

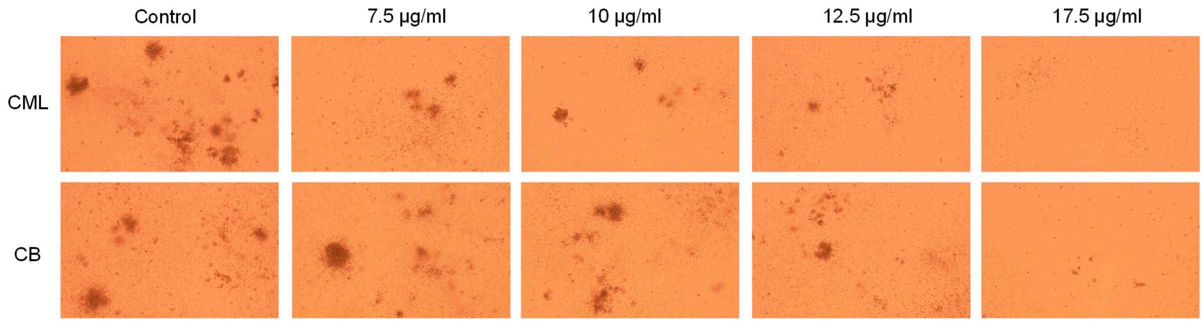

Supplementary Figure S4: Representative colony formation images in BP-CML and CB CD34⁺ cells treated with GI.

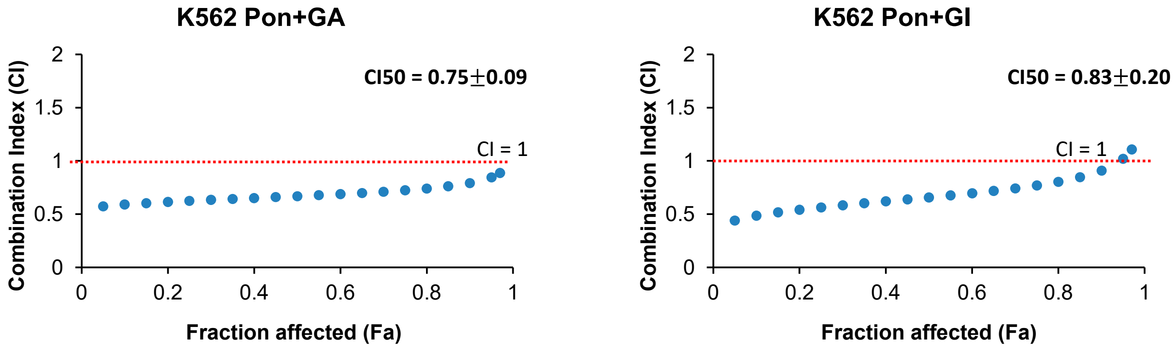

Supplementary Figure S5: GI and GA exhibit synergistic inhibitory effects with ponatinib in K562 cells. Combination index (CI) was calculated using the method of Chou and Talalay and CI < 1 indicates synergism, CI = 1 is additive and CI >1 suggests antagonism. GI and GA demonstrated synergy with ponatinib in K562 cell line with CI<1 at both 50% growth inhibition. Pon: ponatinib. All data are representative of the mean and SD of at least three independent experiments.

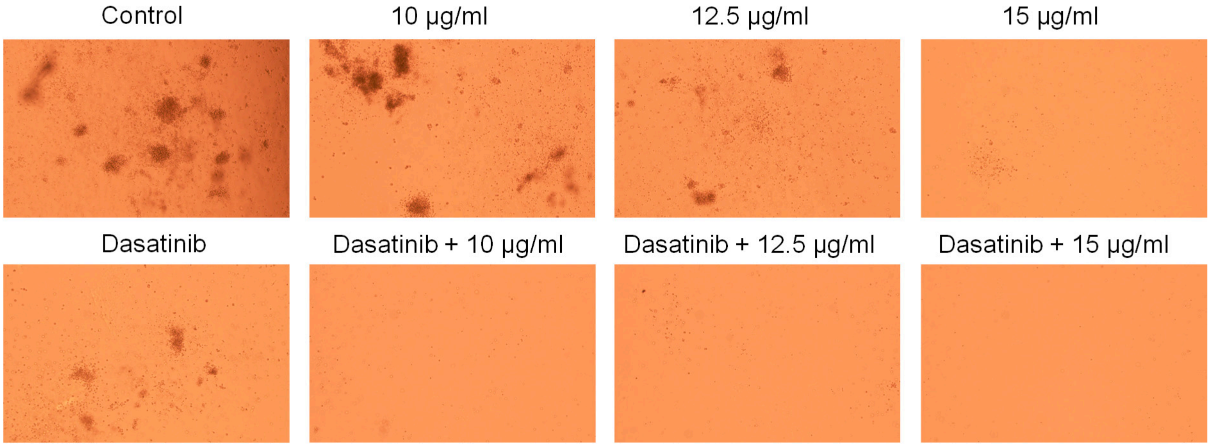

Supplementary Figure S6: Representative colony formation images in BP-CML CD34^{+} cells treated with GI or dasatinib alone, or the combination of GI and dasatinib.

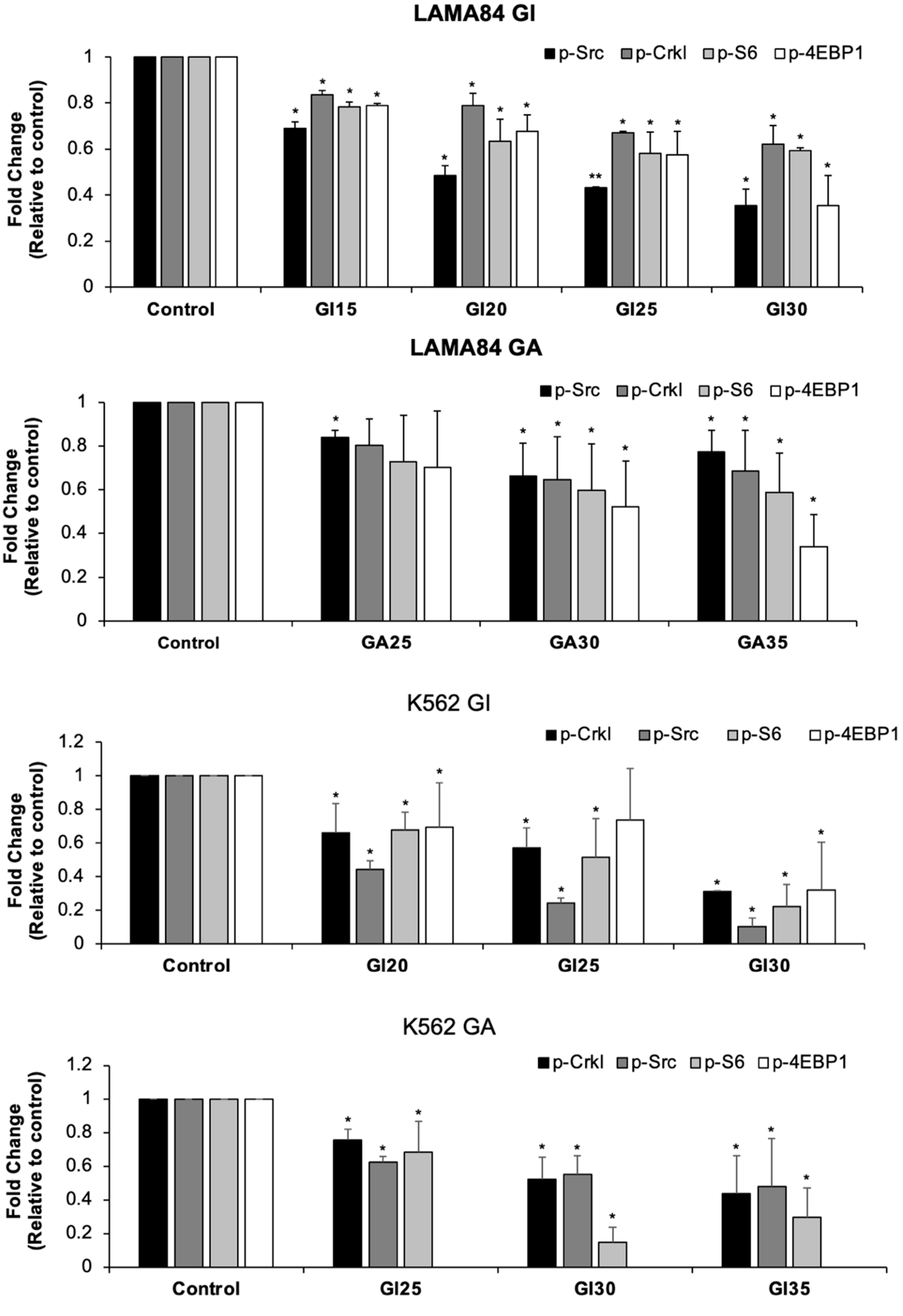

Supplementary Figure S7: Densitometries of phosphorylated proteins against their respective total protein were obtained via ImageJ and normalized against β-actin. Bar graphs are then plotted using fold change of treated samples against control. (* indicates $p < 0.05$).

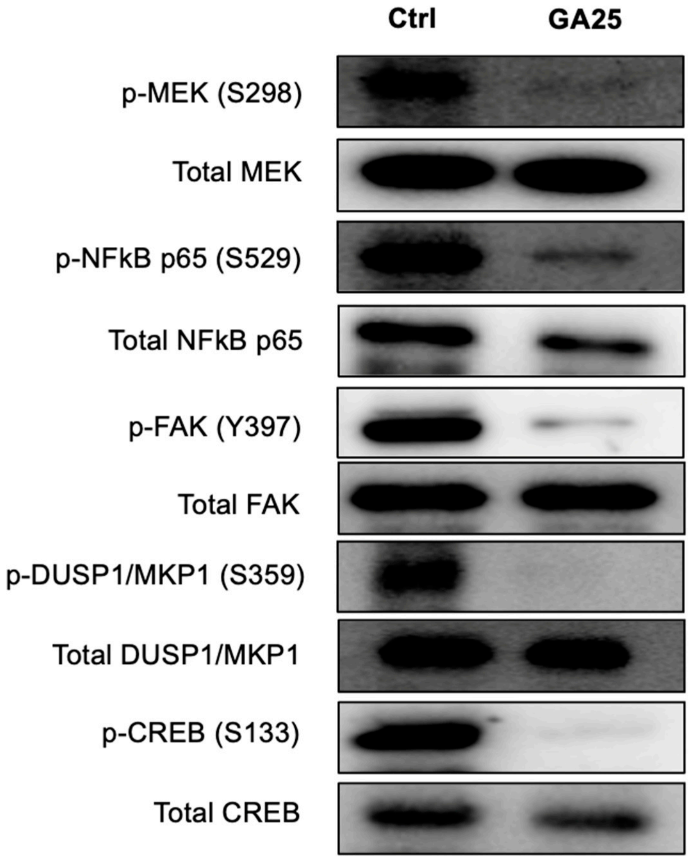

Supplementary Figure S8: Western immunoblotting of MEK and FAK were conducted as secondary validation for phosphoprotein profiling results in K562 cells.

Patient Number	BCR::ABL1 mutation	BCR::ABL1 overexpression	TKI-resistance	Karyotype
BP-CML#3	E453K	No	Imatinib, nilotinib	Not available
BP-CML#5	None	Yes	Imatinib	45,XX,t(3;3)(q21;q26.1),-7,t(9;22)(q34;q11.2)[20]
BP-CML#6	None	No	Imatinib	46,XY,inv(3)(q21q26.2),t(9;22)(q34;q11.2)[20]
BP-CML#7	E255V	No	Imatinib	46,XY,t(7;9;22)(q36;q34;q11.2)[1]/46,idem,der(3)t(3;21)(q26.1;q11.2),add(21)(q11.2)[19]

Supplementary Table S1: BP-CML Patients' information.

LAMA84 IM+GI					LAMA84 IM+GA				
IM only (uM)	IM only Fa	GI only (ug/ml)	GI only Fa	IM+GI Fa	IM only (uM)	IM only Fa	GI only (ug/ml)	GI only Fa	IM+GI Fa
0.001172	0.034	0.054688	0	0.004	0.001172	0.071	0.054688	0.035	0.050634
0.002344	0.055	0.109375	0.051	0.065	0.002344	0.107	0.109375	0.072	0.090233
0.004688	0.044	0.21875	0.028	0.107	0.004688	0.212	0.21875	0.068	0.075482
0.009375	0.116	0.4375	0.049	0.230	0.009375	0.169	0.4375	0.045	0.111666
0.01875	0.061	0.875	0.070	0.198	0.01875	0.041	0.875	0.076	0.123527
0.0375	0.052	1.75	0.089	0.225	0.0375	0.088	1.75	0.133	0.130809
0.075	0.222	3.5	0.273	0.652	0.075	0.302	3.5	0.241	0.529652
0.15	0.758	7	0.623	0.888	0.15	0.801	7	0.610	0.94107
0.3	0.968	14	0.910	0.956	0.3	0.970	14	0.939	0.977742

K562 IM+GI					K562 IM+GA				
IM only (uM)	IM only Fa	GS only (ug/ml)	GS only Fa	IM+GS Fa	IM only (uM)	IM only Fa	GS only (ug/ml)	GS only Fa	IM+GS Fa
0.003906	0.004	0.109375	0.085	0.041	0.003906	0.018	0.109375	0.011	0.073
0.007813	0.035	0.21875	0.059	0.056	0.007813	0.034	0.21875	0	0.049
0.015625	0.030	0.4375	0.075	0.058	0.015625	0	0.4375	0	0.015
0.03125	0	0.875	0.048	0.042	0.03125	0	0.875	0	0.006
0.0625	0.030	1.75	0.108	0.070	0.0625	0.013	1.75	0	0.044
0.125	0.060	3.5	0.204	0.242	0.125	0.019	3.5	0.040	0.086
0.25	0.323	7	0.763	0.898	0.25	0.146	7	0.215	0.309
0.5	0.781	14	0.986	0.984	0.5	0.605	14	0.710	0.851
1	0.864	28	0.990	0.983	1	0.756	28	0.979	0.964

Supplementary Table S2: Fraction affected values of each treatment in CML cells.

	Antibody List		Signal Ratio (Phospho/Total)		Fold Change (G/C)
			Control (C)	Gallic acid (G)	
1	CDK1/CDC2 (Phospho-Thr14)	CDK1/CDC2 (Ab-14)	3.02	0.86	0.28
2	Rel (Phospho-Ser503)	Rel (Ab-503)	0.95	0.28	0.29
3	Raf1 (Phospho-Tyr341)	Raf1 (Ab-341)	1.55	0.50	0.33
4	PLCG2 (Phospho-Tyr753)	PLCG2 (Ab-753)	0.82	0.28	0.35
5	NMDAR2B (Phospho-Tyr1472)	NMDAR2B (Ab-1472)	0.18	0.06	0.36
6	MEK1 (Phospho-Ser298)	MEK1 (Ab-298)	4.85	1.83	0.38
7	Src (Phospho-Ser75)	Src (Ab-75)	0.84	0.32	0.38
8	PLCG2 (Phospho-Tyr1217)	PLCG2 (Ab-1217)	2.67	1.04	0.39
9	NFkB-p65 (Phospho-Ser529)	NFkB-p65 (Ab-529)	7.62	3.1	0.41
10	FAK (Phospho-Tyr397)	FAK (Ab-397)	1.07	0.45	0.42
11	PKC pan activation site (Phospho)	PKC pan activation site	2.90	1.28	0.44
12	PAK3 (Phospho-Ser154)	PAK3 (Ab-154)	1.17	0.53	0.45
13	CREB (Phospho-Thr100)	CREB (Ab-100)	1.41	0.64	0.46
14	MKP-1 (Phospho-Ser359)	MKP-1 (Ab-359)	0.74	0.34	0.47
15	Cyclin D3 (Phospho-Thr283)	Cyclin D3 (Ab-283)	1.26	0.60	0.48
16	STAT2 (Phospho-Tyr690)	STAT2 (Ab-690)	3.79	1.87	0.49
17	CREB (Phospho-Ser133)	CREB (Ab-133)	15.43	7.98	0.52
18	STAT4 (Phospho-Tyr693)	STAT4 (Ab-693)	19.83	10.28	0.52

Supplementary Table S3: List of proteins from phosphoprotein array results included for IPA. Fluorescence signals for each antibody were obtained and a ratio computation was used to measure the extent of protein phosphorylation: phosphorylation ratio = signal of phosphorylated protein / signal of unphosphorylated protein. The extent of protein phosphorylation was compared between GA treated and untreated samples. The background signal was defined as signal of negative control (BSA) and used as a reference to identify true signals.
